# Qualitative Analysis of Round-Table Discussions on the Business Case and Procurement Challenges for Hospital Electronic Prescribing Systems

**DOI:** 10.1371/journal.pone.0079394

**Published:** 2013-11-19

**Authors:** Kathrin M. Cresswell, Ann Slee, Jamie Coleman, Robin Williams, David W. Bates, Aziz Sheikh

**Affiliations:** 1 The School of Health in Social Science, The University of Edinburgh, Edinburgh, United Kingdom; 2 eHealth Research Group, Centre for Population Health Sciences, The University of Edinburgh, Edinburgh, United Kingdom; 3 School of Clinical and Experimental Medicine, University of Birmingham, Edgbaston, United Kingdom; 4 Institute for the Study of Science, Technology and Innovation, The University of Edinburgh, Edinburgh, United Kingdom; 5 Department of Medicine, Brigham and Women's Hospital, Division of General Internal Medicine and Primary Care, Boston, Massachusetts, United States of America; 6 eHealth Research Group, Centre for Population Health Sciences, The University of Edinburgh, Edinburgh, United Kingdom; 7 Division of General Internal Medicine and Primary Care, Brigham and Women's Hospital/Harvard Medical School, Boston, Massachusetts, United States of America; University of Groningen, University Medical Center Groningen, Netherlands

## Abstract

**Objectives:**

There is a pressing need to understand the challenges surrounding procurement of and business case development for hospital electronic prescribing systems, and to identify possible strategies to enhance the efficiency of these processes in order to assist strategic decision making.

**Materials and Methods:**

We organized eight multi-disciplinary round-table discussions in the United Kingdom. Participants included policy makers, representatives from hospitals, system developers, academics, and patients. Each discussion was digitally audio-recorded, transcribed verbatim and, together with accompanying field notes, analyzed thematically with NVivo9.

**Results:**

We drew on data from 17 participants (approximately eight per roundtable), six hours of discussion, and 15 pages of field notes. Key challenges included silo planning with systems not being considered as part of an integrated organizational information technology strategy, lack of opportunity for interactions between customers and potential suppliers, lack of support for hospitals in choosing appropriate systems, difficulty of balancing structured planning with flexibility, and the on-going challenge of distinguishing “wants” and aspirations from organizational “needs”.

**Discussion and conclusions:**

Development of business cases for major investments in information technology does not take place in an organizational vacuum. Building on previously identified potentially transferable dimensions to the development and execution of business cases surrounding measurements of costs/benefits and risk management, we have identified additional components relevant to ePrescribing systems. These include: considerations surrounding strategic context, case for change and objectives, future service requirements and options appraisal, capital and revenue implications, timescale and deliverability, and risk analysis and management.

## Introduction

Hospital electronic prescribing (henceforth referred to as ePrescribing) systems are being implemented by healthcare organizations in an attempt to improve the safety, quality, and efficiency of the medication use process [1]–[4]. In the United Kingdom (UK), these are commonly understood as systems designed to facilitate the processes of medication prescribing, ordering, transmitting, dispensing, administering, and monitoring.

Such systems are being increasingly considered and implemented in much of the economically-developed world, especially in the United States (US), where computerized prescribing in hospitals is a key requirement in achieving meaningful use [5]. The pace of implementation has been slower in other countries – including the UK – but the challenges faced are often similar. For example, implementations are often associated with significant changes to organizational functioning and ways of working [6], [7].

As with any large organizational change initiative involving a major financial outlay, business cases are utilized to outline the underlying reasoning for ePrescribing implementations, including expected investments, benefits and timeframes [8]. This typically also includes the justification for desired changes tailored to individual organizational factors and is sometimes presented as an argument to obtain management commitment for the desired change [9]. However, variations in organizational contexts and needs complicate work in this area [9], this being compounded by a lack of robust empirical efforts systematically addressing key concepts and processes [9], and limited experience of adapting business cases over longer periods of time [10].

At present, decisions are often largely based on anticipated direct financial savings (or proxies to these such as improved efficiency and safety), which are then weighed against the costs of implementation or of achieving such improvements through other means. If the anticipated benefits outweigh the costs, the assumption is that the hospital will become more efficient. Business cases in the UK typically follow a specific format and this same format is used within the National Health Service (NHS) [10]. For example, the NHS Technology Adoption Centre in the UK, a national governmental body tasked with helping healthcare organizations to implement technological change, suggests core components of a business case (Table 1) [11].

**Table 1:** Core components of a business case [11].

Component
Executive Summary
Strategic Context
Case for Change
Objectives
Future Service Requirements
Options Appraisal
Capital Implications
Revenue Implications
Preferred Option
Affordability
Timescale and Deliverability
Risk Analysis and Management
Conclusion

There are however a number of practical challenges to developing ePrescribing business cases within the NHS. These include, but are not limited to: a lack of change management expertise; varying organizational contexts; the relative immaturity of the supplier market with a wide range of available systems with different functionalities (particularly in hospital settings), but rather limited implementation experience of most systems; the complexity of change associated with the introduction of electronic systems which also results in difficulties measuring benefits; and the fact that many systems do not include tools which can help to track benefits after implementation [12]–[14]. Although these applications are nearly always bundled with other types of ordering in the US, such an approach is not yet common in the UK.

Building on earlier work focusing on primary care [7], [15]–[17], we have been commissioned to undertake a national evaluation of hospital ePrescribing systems within NHS England [18]. As part of this work, we are developing a toolkit to support and guide organizations through their implementation journey [19]. In this paper, we present findings from a series of national interactive multi-disciplinary round-table discussions aiming to understand the challenges surrounding procurement and business case development, and identify possible strategies to facilitate associated commercial processes based on the findings.

## Materials and Methods

### Ethics and consent

This work was classed as a service evaluation by the London City & East Research Ethics Committee. We supplied an information sheet to each participant detailing the aims of the study on the day of the event. Written consent to take part was obtained from each participant, comprising a signed consent form. All participants were encouraged to discuss any questions with the research team prior to data collection. The primary goal of the round-table discussions was for participants to exchange experiences, but they were made aware that their anonymous comments would be included in a peer-reviewed, publicly accessible journal.

### Design

We conducted a one-day event including a series of sequential, multi-disciplinary round-table discussions with two parallel groups considering the same topics (see Table 2). Participants were divided into two groups and each group participated in four group discussions (each ∼45 minutes in length amounting to a total of six hours) with designated facilitators to explore different perspectives and dynamics as well as potential ways to align interests [20].

**Table 2:** Key issues explored in the multi-disciplinary round-table discussions.

**Conceptualization and project initiation (in two groups)**
Topic 1: What are the main issues to consider before project initiation?
Topic 2: What are the main aspects involved in project initiation?
**Functional specification and drafting a business case (in two groups)**
Topic 3: How to assess available options in terms of the product?
Topic 4: What are the main challenges involved in drafting a business case?

### Participants

Participants came from a diverse range of stakeholders who had an interest and/or experience in implementing ePrescribing systems in the UK. They were purposefully sampled for maximum variation to include representatives from a variety of sectors including: hospitals that had recently implemented, hospitals in the planning phase, system developers, policy makers, academics, and patients [20].

For sampling, we developed a database of over 400 individuals based in the UK with a potential interest in implementing or adopting ePrescribing systems [18]. This database was based on existing professional networks, contacts from previous related academic research projects, and targeted searches of online conference databases to identify potentially interested delegates. We sent a message with the overall aim of the roundtable discussions to all contacts, inviting interested parties to get in touch with the lead researcher (KC). A total of 47 individuals expressed interest in participating. All of these individuals were sent a draft agenda and asked to add other potentially important items as well as confirm their attendance. All participants that were interested were invited to the event, but we ensured that we had only one representative from each hospital attending in order to maximize the range of perspectives.

We also strategically targeted specific individuals that were from under-represented areas. These were included in our initial database, but did not respond to us inviting initial expressions of interest. For example, we needed to draw on our personal contacts to get representatives from hospitals that had already implemented systems (in order to draw on a range of experiences from hospitals at different stages of implementation), as this event was viewed as being of limited value to such individuals. Overall, 12 participants were recruited through our initial efforts and we invited an additional five participants from our personal contacts.

### Setting

The roundtable discussions took place in Birmingham, UK in October 2012 [18].

### Data collection and handling

Before the roundtable discussions, participants were divided into two groups ensuring maximum representation of different stakeholders in each. Both groups were allocated a moderator (ASl and JC), who led the discussion ensuring focus of discussions and equal input by participants. The day had two main thematic components: 1) conceptualizing and planning implementation (see topics 1 and 2 Table 2); and 2) specifying system functionality and drafting a business case (topics 3 and 4, Table 2).

Each topic was allocated approximately 45 minutes. Each thematic part was followed by group discussion during which the two groups exchanged main areas discussed in a plenary session. The plenary sessions consisted of presentation of overall discussion points by the facilitators, followed by participants discussing similarities and differences between groups and adding additional thoughts/comments.

In the interest of maintaining confidentiality and anonymity, participant professional roles and locations were anonymized by assigning broad categories and numbers.

Each group discussion was audio-recorded and transcribed. In addition, designated researchers (KC and RP) took field notes relating to perceived dynamics and interactions between participants.

### Data analysis

Transcribed data for each topic and group were uploaded into NVivo9 software (a qualitative data analysis software that allows organizing textual data) to facilitate coding. This was initially done along the four topic areas, followed by thematically coding emerging themes inductively [21], [22]. Emerging findings were then discussed within the wider research team leading to the refinement of categories [23]. Areas that were repeatedly identified across different stakeholder groups as either being a subject of tension or agreement were explored in most detail.

We drew on a lifecycle perspective of technology implementation in analyzing the data and conceptualized the business case stage as part of the beginning of the journey towards full ePrescribing implementation [18], [24], [25]. This was complemented by drawing on a theoretical approach that we have developed and refined in previous work to guide data collection and analysis activities [26], [27]. This provides a structure for examining different aspects of the lifecycle of ePrescribing implementation situated within the macro-context of a large, dynamic national health system. Our findings were then organized along the business case components outlined in Table 1.

## Results

Drawing on data from 17 participants, our complete dataset comprised eight audio-recordings lasting six hours and 15 pages of researcher field notes. Participants included nine representatives from hospitals at various stages of implementation, four system developers, two policy representatives, one patient, and one academic (Table 3).

**Table 3:** Participant characteristics.

	GROUP 1	GROUP 2
1	Male	Female
	Policy	Policy
2	Female	Male
	Physician	Pharmacist
	Hospital planning to implement	Hospital planning to implement
3	Male	Female
	Project Manager	Project Manager
	Hospital in process of implementing	Hospital in process of implementing
4	Male	Female
	Pharmacist	Pharmacist
	Hospital planning to implement	Hospital has implemented
5	Male	Female
	Pharmacist	Pharmacist
	Hospital in process of implementing	Hospital has implemented
6	Male	Female
	System developer	Nurse
		Hospital planning to implement
7	Male	Male
	System developer	System developer
8	Male	Male
	Academic	System developer
9	Female	
	Patient	

Overall, benefits of systems that were viewed as realistic amongst participants included the following:

- Reductions in prescribing errors and improved patient safety through decision support functionality and legibility;
- Improved quality of care through improved access to information and information flow;
- Improved guideline implementation and compliance;
- Secondary uses of data (e.g. audits, among many others).

We identified the following overarching themes:

- Strategic context: considering the implementation of ePrescribing as part of a wider organizational strategic development.
- Case for change and objectives: developing and maintaining relationships between customers and system suppliers throughout procurement and implementation.
- Future service requirements and options appraisal: choosing systems based on functional specifications and drawing on experiences of other hospitals.
- Timescale and deliverability: planning the change whilst maintaining strategic flexibility to respond to changing needs and environments.
- Timescale and deliverability: separating “wants and aspirations” from organizational needs.

These are summarized in Table 4 with detailed sub-themes and will be discussed in turn with supporting illustrative quotes from the data.

**Table 4:** Themes and sub-themes emerging from the data.

**Strategic context: ePrescribing as part of a wider organizational strategy**
- High-level drive and support from senior organizational stakeholders
- Inter-disciplinary involvement
- ePrescribing as an essential component of the overall organizational information strategy
- Organizational information strategies and associated ePrescribing system choices
**Case for change and objectives: developing and maintaining relationships between customers and system suppliers**
- Relationship building before and throughout the implementation journey
- A long-term partnership characterized by mutual trust but restrained by commercial relationships
- Sharing experiences of systems and suppliers through reference sites, supplier days and informal networks
**Future service requirements and options appraisal: system choice through functional specifications and shared experiences**
- Systems choice guided by functional specifications and networking with sites that have implemented
- Minimum system functions and outcome based specifications
- Restrictions in system choice and financial restrictions
- Pooling resources and sharing experiences
**Timescale and deliverability: planning the change whilst maintaining strategic flexibility**
- Workflow and process mapping
- Stakeholder engagement
- Investment and resources
- Parallel systems and interoperability
- Composition of the project team
- Journey as opposed to a project
- Changing needs and flexibility in strategy
**Timescale and deliverability: separating “wants and aspirations” from organizational needs**
- Expectations often exceed reality
- Organizational versus individual benefits
- Wishes versus needs

### Strategic context: considering the implementation of ePrescribing as part of a wider organizational strategic development

High-level strategic direction and support from senior organizational stakeholders were viewed as important pre-requisites for implementing ePrescribing systems as they were believed to affect many different aspects of organizational functioning. Senior commitment was perceived to be essential in ensuring the availability of necessary financial resources to support implementation, maintaining project momentum, and coordinating efforts across the organization to improve overall business processes.

*It's a hospital wide system isn't it, it's deployed right across the hospital so in some senses the ownership must sit with the top tier of management within the hospital…it's a system that's going to interconnect with so many other components of the electronic patient record so I think it's got to be owned at that level. (Group 2, Male, System developer)*

Participants stated that ideally this high-level ownership should be characterized by the “hands-on” involvement of senior hospital staff in order to ensure that the implementation remained an organizational priority over time. This was perceived to be facilitated by integrating ePrescribing as an essential component of the overall organizational information strategy.

*…it's part of the information strategy, with the current financial situation it's how it fits into the organizational strategy generally, it's not just IT [information technology] or information it's “is this a priority for the organization? Is it something that is fundamental about how the organization does business?” (Group 1, Male, Pharmacist, hospital planning to implement)*

Participants discussed two different common conceptualizations of this overall organizational information strategy. Firstly, implementation of IT could be viewed as an opportunity to fundamentally change existing ways of working and business processes. This was often stated to be associated with the hardest work, but also the biggest potential benefits. Secondly, an organizational information strategy could be viewed as a project designed to enhance existing business processes. This approach was seen to require less radical change and be easier to “sell” to stakeholders as changes to individual ways of working were perceived to be less fundamental. Participants stated that benefits would be quicker to realize, but also tended to be more limited than those associated with process changes in radical business process re-design.

*Most hospitals do not perceive their hospital based project to be a way of radically changing the way they work as an organization or treat patients as a process. They see it as an initiation of an IT system or a new theatre or a new hospital wing or whatever project it is they're doing, it's an enhancement to existing ways of doing things rather than an opportunity for radical change. So when you think about strategy it really depends on what level it's set and what you perceive strategy to be…(Group 2, Male, System developer)*

These two ways to conceptualize organizational information strategies were associated with different ePrescribing system choices: integrated systems were more commonly seen to be part of a fundamental business change strategy, whilst “stand-alone” ePrescribing systems (prescribing administration systems or those integrated with a pharmacy stock control system) were seen to be associated with strategies to enhance existing processes. As a result, there were therefore at least two different types of business cases for ePrescribing systems, each with very different resource implications, ownership and expected benefits.

### Case for change and objectives: developing and maintaining relationships between customers and system suppliers throughout procurement and implementation

No matter what the organizational strategy, all stakeholders agreed that establishing and maintaining good relationships between hospitals and system suppliers was a pre-requisite to any successful implementation journey. However, this was not always encouraged and was hindered by existing tendering processes that tended to promote “arm's length” relationships in the early stages. Relationship building with a range of suppliers should ideally start long before the signing of a formal business case, in order to understand what systems and system suppliers offered and how products would fit in with organizational processes and future plans.

The recognition that both suppliers and customers were entering a long-term working partnership which should ideally be characterized by open and honest dialogue was critical. In doing so, both should have similar underlying visions and values (as far as possible with one public service body and one commercial entity), as well as a common desire to succeed and develop together over time to meet new emerging challenges.

*…most system vendors should be saying that they want to work in partnership with the NHS…it's not just about a two year thing or a three year project it's a longer term project so as part of that you have to also make sure that any organization you're working with is also aligned to your organizational values…or just as wedded to actually achieving a successful implementation at the end of it…(Group 1, Male, System developer)*

Trust was viewed to be a necessary component of the relationship between customers and suppliers. Examples here included trust in that both parties were getting benefits from working together and that nobody was being “ripped off”, but also trust on the part of the customer that the necessary functionality would be delivered by the developer.

*…the process doesn't really allow you to get to know the suppliers well enough to know if you can trust them and vice versa…that long term relationship with the system supplier and it colors everything so…the mutual trust that you have so that suppliers don't feel they're getting ripped off and we don't feel we're getting ripped off. Where things have not gone well often it comes back to that and it's a war of attrition. (Group 1, Female, Physician, Hospital planning to implement)*

An important aspect of engagement activity between suppliers and customers was viewed to be open and honest discussion of expectations and system capabilities, including potential limitations and risks, on both sides. Ideally, this should be coupled with system demonstrations/networking in/with sites that had already implemented relevant systems and going through test scenarios incorporating specific organizational needs. This was perceived to help hospitals apply system functionalities to their own organizational processes and see the potential benefits of a fully functioning system.

In addition to formal reference sites recommended by suppliers (which might be expected to give a ‘rosy’ picture of benefits and challenges), hospital staff stated that relying on informal personal and professional networks was important to obtain insights into the challenges experienced with a particular supplier, and also to explore potential benefits and approaches to successfully working with them. Informal networks were generally supported as they were perceived to have a positive influence on system choice, but unanticipated issues were mentioned in relation to formal networks.

*But then that's a challenge because if those reference sites receive certain discounts or support because they're prepared to be reference sites…does that then slightly temper what they may or may not be prepared to say? (Group 2, Female, Pharmacist, hospital has implemented)*

### Future service requirements and options appraisal: choosing systems based on functional specifications and drawing on experiences of other hospitals

Participants stated that actual systems choice should be guided by functional specifications. NHS Connecting for Health, an arms-length governmental body charged with overseeing the implementation of national eHealth systems in England, had done important groundwork in this respect, but the national strategy was abolished in 2011 along with NHS Connecting for Health [28]. Participants argued that such functional specifications should be based on local needs, and by visiting sites that had already implemented in order to gain an insight into the practical use of the system for clinical management as well as processes associated with implementation and planning. However, hospital representatives stated that the difficulty surrounding learning from other sites was the fact that every organizational starting point and needs differed significantly; for example how a new system would inter-operate with existing systems and management processes. Suppliers on the other hand felt that hospital processes did not vary as much as some hospital stakeholders thought.

All agreed that the first step relating to systems choice should be an assessment of the current status quo (in terms of existing processes and systems), a mapping-out of the desired future state, and consideration of the steps to get there. An outline of minimum system functions (including safety standards) and desired outcomes was viewed to be a necessary part of this process. Outcome-based specifications (defined as specifications based on the functional requirements for the proposed development without addressing how those outputs may be achieved) were seen as a means for customers and suppliers to achieve a common goal, but it was also acknowledged that such specifications were open to interpretation and could mean different things to different stakeholders.

*…that's the difference though is that the OBS [outcome-based specification] is what the outcome is, not how it does it. Because…you can write on the spec it must have a single sign on but the OBS will be that you must have security. So that's why the OBS I would think is a better way…because it's not that level of detail but that's uncomfortable for the [hospital] because I want it to exactly, to do this rather than it needs to be able to deliver this. (Group 1, Male, Pharmacist, hospital planning to implement)*

Similarly, detailed functional specifications were seen as necessary to detail specific processes, but participants also recognized the inherent tension between tight functional specifications (which were often aspirational) and associated restrictions in systems choice, suppliers and innovation which may have a detrimental effect on the tendering process.

*…there are different ways to procure things nowadays and therefore a lot of the procurement approach is going to be things like restricted energies where you're producing a very tight specification so you're not necessarily going out to do anything like competitive dialogue…(Group 1, Male, Policy)*

*…on that supplier day there were only two suppliers and that was because of the detailed nature of our functional spec…at the beginning and so…if you want to see the full range of what's available you need to broaden your [specification]… (Group 1, Male, Pharmacist, hospital in process of implementing)*

System choice and writing functional specifications were further perceived to be restricted by a limited knowledge of existing system functionality amongst the NHS as well as a lack of resources to actively seek out suppliers and spend time at reference sites.

*…there's a couple of systems that have lots of…sites using them but most systems have very little experience and use and it's quite hard to equate that. And a lot of, it's very hard to go and see all of those systems and everything that's out there because the local [hospital] can't afford to send you off to do all of that. (Group 2, Female, Project Manager, hospital in process of implementing)*

In order to address the issue surrounding financial restrictions, hospital representatives suggested that existing resources (e.g. relating to functional specifications, hazard assessments, implementation progress) should be pooled wherever possible and that experiences and lessons learned as well as potential areas of risk associated with different systems should be formally shared across hospitals. Such sharing of experiences was however either non-existent, leading to efforts being duplicated across locations, or was taking place informally, leading to unequal access to information across the health service.

### Timescale and deliverability: planning the change whilst maintaining strategic flexibility to respond to changing needs and environments

Throughout the planning and implementation stages several practical issues to be included in the business case related to workflow and process mapping, stakeholder engagement, investment and resources, parallel paper and electronic systems and interoperability, and the composition of the project team.

User workflow and process mapping was viewed as necessary to provide a baseline from which the organizational strategy for change could be developed. Participants argued that organizations that were clear about current and expected organizational changes to improve efficiencies tended to find it easier to choose an appropriate system, plan for required resources, and keep to the deliverables.

*…given that prescribing is the most common intervention…it's going to come in at a multiple number of points in a workflow, not necessarily just a prescribing workflow but admissions all the way through. And I think if that was done more often, and in the procurements that I've seen where organizations are very clear on how and what they want to deliver it certainly helped them challenge the vendors to actually deliver more at an earlier stage and be clear on what needs to come out of a solution. (Group 1, Male, System developer)*

Most hospital stakeholders were of the opinion that the amount of resources required was often under-estimated when planning for implementation. This was most commonly perceived to be associated with implementation-related costs (as opposed to the capital needed to buy the system itself).

*… what we underestimate is the amount of money we have to spend on implementation. We all think when you buy the system that's it…actually buying the system is just the beginning and I think we constantly underestimate that…(Group 1, Female, Physician, hospital planning to implement)*

*…the other two big things, one was interfacing, developing interfaces and making things work between different systems and the other one was the amount of operational time that's needed, so a lot of clinical pharmacy time that you wouldn't include in your project costs and probably nursing and clinical time. (Group 2, Female, Project Manager, hospital in process of implementing)*

In addition, and despite acknowledging that the business case should be used as the central project planning vehicle, participants emphasized that changing needs and environments required a certain degree of flexibility. Those that had already implemented for example stated that their needs had changed throughout different stages of the journey.

*… from where we started two and a half years ago it has changed dramatically and the emphasis has changed and the priorities have changed…(Group 1, Male, Project Manager, hospital in process of implementing)*

Some also stated that financial aspects of the business case may need revision and refinement as more in-depth knowledge is developed over time.

*…you should write just a business case for the next stage which is your procurement so it might start to touch on a lot of the areas but it wouldn't necessarily talk about financials because you wouldn't know about those so you couldn't therefore do a proper financial business case…So you'd probably look at it in terms of…reduction of clinical risks and…the sort of non-financial things but I don't know how you could do a financial business case until you've done some of the later work so the two sort of things need to almost go along side by side. (Group 1, Male, Project Manager, hospital in process of implementing)*

### Timescale and deliverability: separating “wants and aspirations” from organizational “needs”

Managing expectations was mentioned frequently, with the perception amongst many that most organizational stakeholders over-estimated actual benefits and underestimated potential adverse impacts of ePrescribing systems. This was perceived to be particularly true in relation to costs and individual workloads for end-users as these would, despite overall business gain, increase for some end-users with implementation.

*…so the benefit is not for the person who carries out the work so there's a transfer of work. Standards improve, people have to do more work and the people who are doing the work are not the ones who see the immediate benefit. (Group 1, Female, Physician, hospital planning to implement)*

*…in our [hospital] absolutely, it was money and it was seen as a potential way of saving, you know, saving money….we haven't been able to show that yet…(Group 1, Male, Pharmacist, hospital in process of implementing)*

Currently, it was felt that especially among users, expectations of what the system would be able to do often far exceeded reality, with limited appreciation of the risks.

*…part of the problem we had was that at the initial stages the expectations of the clinicians and the nurses and the pharmacists of the things that, the system we bought was going to be able to do was in no way matched up to what we've got.…(Group 2, Female, Pharmacist, hospital has implemented)*

*… we came out with a list, a huge great big long wish list and then have ended up with a system that doesn't achieve any of those things and so the expectations are wildly different. The expectations of our clinicians, a lot of them is that this is going to…solve lots and lots of problems and it isn't, and actually that makes the problems that it does cause…more acute to them because that's balancing the see-saw even further in the other direction. (Group 1, Male, Pharmacist, hospital in process of implementing)*

In line with these high expectations, it was felt that many hospital stakeholders had developed a “wish list” of system features that developers were unable to fulfill. Therefore, it was argued that organizations should conceptually separate “wants and aspirations” from organizational needs, although this is clearly not black and white. However, better prioritization could result in more productive working relationships with developers as well as a more efficient management of user expectations.

*There's like the must haves, the icing on the cake and things that would be nice to come afterwards. (Group 2, Female, Project Manager, hospital in process of implementing)*

*There are certain [features] which are very much aspirational, there are certain ones which are must haves and there are certain ones that are a bridge between those two ends of the spectrum and you need to be able to migrate from the must haves into the aspirational wants if they're still relevant. (Group 2, Male, System developer)*

It was further acknowledged that there could be adverse effects associated with implementations and that these would need to be anticipated, measured and managed. For instance, hospitals were often forced to make ad-hoc and unanticipated system changes due to systems presenting new unexpected safety risks:

*…the spec said it ought to have everything else but it doesn't and during the roll out we've had to make difficult decisions about changing things from a safety point of view as a result of things that have happened as we've rolled out. And you need the ability to do that and to respond to those quite quickly…(Group 1, Male, Pharmacist, Hospital in process of implementing)*

## Discussion

We convened a wide variety of national stakeholders from different professional backgrounds to understand some of the challenges surrounding procurement and business case development relating to ePrescribing, and identify possible strategies to enhance the efficiency of these processes. Key findings were that such organizational change initiatives should ideally be viewed as a fundamental aspect of a wider organizational strategy, and characterized by long-term partnerships within and between the NHS and suppliers. Planning of financial resources and associated local needs is vital, although the journey must also be characterized by some strategic flexibility as new needs emerge and technologies develop over time. This also requires a realistic appreciation of the benefits and trade-offs of such systems. Key benefits were viewed as including: reductions in prescribing errors and improved patient safety through decision support functionality and legibility; improved quality of care through better access to information and information flows; improved compliance with clinical guidelines; and innovative secondary uses of data [29]. We summarize possible efficiency enhancement strategies emerging from this work in Table 5. Surprisingly, improved workflows and efficiency were not cited as benefits, although observed in previous work [8]. This may be due to the fact that systems discussed were still at relatively early stages of implementation with limited scope for customization.

**Table 5:** Possible efficiency enhancement strategies emerging from this work.

**Strategic context**
Developing a clear roadmap of how ePrescribing fits in with the wider long-term organizational IT strategy. This should involve detailed mapping of required input from and engagement of a wide range of organizational stakeholders beyond the pharmacy department. It should also include a realistic assessment/planning of anticipated benefits and a recognition that these are likely to materialize in the medium- to long-term.
**Case for change and objectives**
System choice needs to emerge from this strategy and should be informed by a detailed appreciation of the needs of different stakeholders. Ongoing evaluation of these needs through continuous engagement both prior to and after procurement is therefore vital. This requires an assessment of potential future scenarios relating to both organizational vision and existing/future system functionality, as well as an assessment of strategic alignment between organizational strategies and available systems.
**Future service requirements and options appraisal**
Longer-term relationships with suppliers can be greatly facilitated by discussing expectations on both sides in advance and agreeing on a common goal. This should involve assessing desirable and essential functionality, but also potential technical and financial constraints. Shared risk registers tackling areas of particular importance (e.g. resources, interoperability, changing needs) can be a good way of achieving this.
Networking with other healthcare organizations is essential. Designated individuals should be identified to frequently attend information sharing events and conferences in order to make and maintain important contacts.
**Timescale and deliverability**
Tracking of system benefits needs to be conducted throughout planning, implementation and routine use. This should involve baseline measurements as well as assessments of short-, medium- and longer-term benefits.

When examining the literature surrounding information systems more generally, there are several existing models postulating the importance of the alignment (or fit) between business/organizational processes and IT systems [30]–[33]. Our findings resonate with this body of work as organizational change initiatives involving IT systems are increasingly being considered as an intricate feature of wider organizational strategies and processes. Our work has also illustrated the important inter-relationships between organizational and technical considerations [32], [33]. For example, whilst strong leadership is likely to facilitate change [30], this also needs to be cognizant of emergent technical opportunities and choices and have an appreciation of how to embed these systems into everyday care processes [33].

Others have evaluated a number of specific issues around ePrescribing applications, including success factors for implementation and cost-effectiveness [8], [19], [34], impacts on medication safety and potential to cause harm [35]. The UK is now at a key stage, as hospitals around the country are considering embarking on this journey [6], [36].

While the considerations raised in the discussions we evaluated were not exhaustive, they provide a starting point for hospitals that are planning to implement relevant systems. In doing so, they are building on the important work conducted by NHS Connecting for Health in 2009, outlining a list of questions that hospitals should ask themselves before venturing forth with implementing ePrescribing systems [28]. We offer a set of revised questions based on the results of the present work in Table 6.

**Table 6:** Questions that hospitals should ask themselves before venturing forth with implementing ePrescribing systems.

**Strategic context**
1. What is the overall organizational information strategy and how does ePrescribing fit into this in the short-, medium- and long-term?
**Case for change and objectives**
2. Which system and which supplier fits best within this information strategy?
3. Has the implementation necessary inter-disciplinary buy-in across the organization?
**Future service requirements and options appraisal**
4. How do hospital and supplier visions and short-, medium- and long-term strategies align?
5. Has anyone else implemented this system and what are their experiences? Ideally share experiences on an on-going basis.
6. What are current organizational processes, what is the desired future state (in the short-, medium- and long-term), and what steps need to be taken to get there?
7. What functionality can local resources realistically buy now and in the future? This will also require accounting for additional staff, infrastructure, interfacing etc.
**Timescale and deliverability**
8. What is essential functionality and what is desirable? This should involve assessing organizational and individual needs of each professional stakeholder group.
9. What are realistic organizational and individual benefits?
10. What changes to systems, needs and strategies can be expected in the future?

Care however needs to be taken in attempting to transfer findings from this study to other contexts. This is because prior work has highlighted the highly contextualized nature of developing business cases and procurement processes of major IT systems in complex organizational environments [9]. Consequently, due to variations in need, demographics and strategies, healthcare organizations are faced with having to translate these somewhat abstract general concepts into concrete organizational approaches. That said, there appear to be some transferable dimensions to the development and execution of business cases, which the information systems literature has begun to identify. These include information on benefits, costs and risks, as well as methods of measuring impacts [10], [37]–[39], and can be used to support deliberations surrounding making the case for change, assessing capital and revenue implications, and risk analysis and management (Table 1). More specifically in relation to ePrescribing systems (Table 1), we have attempted to identify potentially transferable lessons in relation to strategic context (by outlining prominent organizational strategies), case for change and objectives (by beginning to gain insights into realistic benefits that can be expected), future service requirements and options appraisal (both of which can be facilitated by collaboration and networking with other organizations and suppliers), capital and revenue implications (by assessing necessary investments and resources), timescale and deliverability (by maintaining a realistic expectation of benefits and work required), and risk analysis and management (through stakeholder engagement and meaningful work process mapping).

A key strength of our efforts relates to the balance between conducting methodologically sound and theoretically informed research with high policy and clinical relevance. The work is likely to be generalizable across NHS settings, but may have more limited applicability to international health systems due to variations in context. Nevertheless, there may be some transferable lessons, particularly in relation to very large healthcare systems, such as the Veterans Health Administration or Kaiser Permanente in the US, or in relation to other health technologies. We have brought together stakeholders from different backgrounds and encouraged and facilitated frank discussion and debate in this important, but hitherto largely neglected area. Naturally, there are also limitations emerging from this balance. For example, our facilitators were not trained qualitative researchers, but practitioners with established credibility within the relevant stakeholder groups, having previously worked within NHS Connecting for Health. Similarly, the fact that discussions were recorded may have inhibited some participants from disclosing important information. It further became apparent that open discussion was somewhat inhibited in groups where system suppliers and staff from hospitals installing/using the same system were placed in one discussion group, as certain statements were feared to be perceived as potential criticism by some. In addition, the participants may not have recognized all potential benefits, and may not have been able to anticipate what their relative contributions would be. More generally, the number of participants was small, which may have resulted in limited insights relating to the range of different roles. However, the relatively intimate format also allowed all participants to contribute to discussions and ensure that their views were explored in depth.

Overall, our results have illustrated that implementation of ePrescribing systems should not take place solely within the pharmacy department – as is often perceived to be the case by some organizational stakeholders in the UK [13]. This is because these systems have a significant impact on provider time and they are intricately associated with wider organizational processes and strategies that involve changing workflows for a range of hospital professionals. Organizations need to incorporate the implementation of ePrescribing within their strategic planning from the start in order to ensure an integrated approach to improving safety and efficiency. Equally, extra-organizational groupings such as suppliers and other implementing organizations play an important role in the realization of desired benefits and system development over time. They therefore need to play a central role in organizational activities surrounding the preparation of business cases for ePrescribing systems. Networking with other implementing sites and suppliers can not only help to disseminate lessons learned, but also to ensure that systems are being developed in collaboration and refined accordingly to suit the health system as a whole.

Our findings reinforce the assumption that technical, human and organizational dimensions are situated within and influenced by a larger environment including a web of other healthcare organizations, industry stakeholders (e.g. system suppliers), the media, governmental bodies and associated policy, professional groups, and the general economic landscape [12], [40]–[43]. The literature shows how these associated factors can shape the implementation of technology in important ways, and our work highlights the need to extend these considerations to include the early stages of the technology lifecycle, namely those that relate to conception and planning of the technological change.

## Conclusions

Developing business cases to justify investments is a central component of planning for the implementation of ePrescribing systems. The area is ripe for discussion and debate as the number of English hospitals preparing themselves for procurement is steadily increasing [36]. In order to facilitate efficiency and maximize existing expertise, it is vital that business cases are built on a solid foundation and that lessons are shared between settings wherever possible. Building on previously identified potentially transferable dimensions to the development and execution of business cases surrounding measurements of costs/benefits and risk management, we have begun to identify components of ePrescribing system business cases that may facilitate this sharing. The UK will also have to consider the experiences of other countries around issues such as whether to link ePrescribing to other types of ordering and what the potential benefits of implementation of this technology will actually be.
